# Calculating expected DNA remnants from ancient founding events in human population genetics

**DOI:** 10.1186/1471-2156-9-66

**Published:** 2008-10-17

**Authors:** Andrew Stacey, Nathan C Sheffield, Keith A Crandall

**Affiliations:** 1 Department of Statistics, Brigham Young University, Provo, UT 84602, USA; 2 Department of Biology, Brigham Young University, Provo, UT 84602, USA; 3 Battelle Center for Mathematical Medicine, Nationwide Children's Hospital, the Ohio State University, 700 Children's Drive, Columbus, OH 43205, USA

## Abstract

**Background:**

Recent advancements in sequencing and computational technologies have led to rapid generation and analysis of high quality genetic data. Such genetic data have achieved wide acceptance in studies of historic human population origins and admixture. However, in studies relating to small, recent admixture events, genetic factors such as historic population sizes, genetic drift, and mutation can have pronounced effects on data reliability and utility. To address these issues we conducted genetic simulations targeting influential genetic parameters in admixed populations.

**Results:**

We performed a series of simulations, adjusting variable values to assess the affect of these genetic parameters on current human population studies and what these studies infer about past population structure. Final mean allele frequencies varied from 0.0005 to over 0.50, depending on the parameters.

**Conclusion:**

The results of the simulations illustrate that, while genetic data may be sensitive and powerful in large genetic studies, caution must be used when applying genetic information to small, recent admixture events. For some parameter sets, genetic data will not be adequate to detect historic admixture. In such cases, studies should consider anthropologic, archeological, and linguistic data where possible.

## Background

In the past 20 years, DNA sequence data and advanced computational techniques have provided an unparalleled resource in the study of human origins[1] and migration[2]. These tools have demonstrated a Pleistocene colonization of America by Asian populations[3,4] and have even prompted calculations of the size of the original human founding populations[5]. Similarly, DNA sequence data have helped demonstrate the dynamics of large human populations such as primitive human migration out of Africa[6], the American migration[3], the Lemba migration in Africa[7], the migratory history of the Baltic States[8], and many others. Researchers have even used the population genetics of human disease vectors to trace human migration events[9]. It may be difficult to underestimate the value genetic data have played and will continue to play on our ability to reconstruct historic population events.

But while sequence data have been used to study many forms of human migration, their utility in the study of small-scale migration is still in question. Research into small migrations like the Norse settlements in Greenland[10], a possible Polynesian migration to the New World[11], the North African Slave migration to America[12], and the pre-Columbian European migration to America[13,14], have traditionally been based primarily on evidence other than DNA sequence information. However, recently, researchers have begun to apply genetic data to these smaller historical migrations and make conclusions about small historic populations using current DNA. For example, DNA information has recently been used to study the small indigenous populations of Tierra del Fuego[15], and to analyze Caucasian admixture in specific African American populations[16,17]. It should be noted that genetic data have been used to study the large Norse migration to Ireland[18], but are an afterthought when researching their short-lived occupation of Canada[19,20].

This raises questions about the utility of genetic data in providing evidence for historic migrations and inferences of unknown past events. While genetic studies can provide considerable information, they are also accompanied by variation and stochasticity. Because of these limitations, even the most complete studies of human populations have been called "not unequivocal"[21] or "sobering"[22] by those conducting the research. Recent reports have also addressed the limited depth of current genetic studies[23], indicating that most studies make conclusions after sequencing less than 1% of subjects' genomes, and sampling only small numbers of a population. Such methods can be especially problematic when dealing with historic admixture events that are very small. The difficulty is a function of the current architecture of genetic studies: researchers sample loci from a group of individuals and categorize individuals into groups based on which alleles they have at the loci tested[24,25]. These categorizations are determined based on the most prevalent or probable genetic markers in an individual's genome. The results of these studies, then, can overlook genetic markers that simply are not sampled, which is common in small admixture events. Additionally, stochastic events can lead to allele fixation and further complicate matters, particularly in small populations. It has been suggested that studies of even the largest migrations should couple genetic information with archeological, anthropological, and linguistic data[26].

As our ability to collect and analyze DNA sequence data increases, understanding the probabilities and variability associated with admixture becomes especially important. In this study, we explore the utility of DNA sequence data in small, recent human migration studies. We use forward-based genetic simulation to explore three questions: 1) what variables contribute to the presence (or absence) of historic markers in today's genomes, 2) how do these variables affect the probability of finding historically admixed DNA in today's populations, and 3) how can studies be designed to maximize information from genetic data? These questions are answered through genetic simulation and a sample size study aimed at suggesting the numbers of subjects and loci that should be sampled to successfully detect small-scale admixture. In our simulations, we assume that migrant allele frequencies are known a priori. The simulations test our ability to detect these known migrant alleles in admixed descended populations. We find that genetic parameters, the stochasticity of genetic drift, and experimental design all play an important role in the ability to find historic DNA in current admixed populations.

## Methods

We used the simuPOP software package for forward-based genetic simulations[27]. In each simulation, a "migrant" population with distinct, known alleles was admixed with a "native" population. We followed the combined population through time and recorded the frequency of migrant alleles at each generation. Because migrant genetic parameters were known a priori, these simulated allele frequencies allow us to assess how parameters affect the ability of detecting migrant alleles in an admixed descendant population. We used a generation time of 23 years as a compromise among differing estimates of human generation times [28-30]. The simuPOP module allows numerous genetic variables to be altered and studied independently. The variables of interest in these initial simulations are basic genetic variables: native population size, migrant population size, mutation rate, time since admixture event, and initial allele frequencies. These variables allow the assessment of the role that population sizes, mutation, genetic drift, and allele frequency have on the amount of migrant DNA present in the admixed population after a number of generations. Our simulations have been designed so that total population sizes are as analogous to effective population sizes (N_e_) as possible. We assume that each individual has an equal expectation of obtaining progeny, that there are equal sex ratios, and that the population remains constant over time[31]. These assumptions allow the population size used in our study to be interpreted as an effective population size, though under some definitions of N_e _our numbers will have different values of N_e _than those assigned. The statistics and results in this study are based on the allele frequencies retrieved from the simuPOP software. We imported these numbers into the R statistical package for numeric and graphical analysis.

In our genetic simulations, we make a number of assumptions about the populations: random mating, absence of selection, no gene flow, and constant population size from time of the migratory event to the present. Actual populations experience some gene flow with neighboring populations[32,33]; however, in our simulations, we do not consider this in an attempt to create a best-case scenario for the migrant allele. If such gene flow did occur, it could only decrease the chances of detecting the migration event by lowering the frequency of the migratory allele in the admixed population. In addition, real populations often experience growth following admixture. However, assuming that the migrant allele is growing at the same rate as the other alleles (random mating), the allele frequency should not be changed directly by population size increase[34], although the effects of drift could become less pronounced as a result of a greater population size. Further studies and simulations using population growth rates may be helpful in addressing the effects of population growth.

### Simulations

Our simulations can be grouped into two separate categories. The first is a series of simulations designed to assess how the parameters mentioned above can influence the presence of historic migrant DNA in today's populations. More concretely, these simulations answer this question: how does each genetic parameter affect the frequency of migrant alleles in an admixed population? Our simulations tested the effect of 4 variables: size of migrant population, size of native population, time since admixture event, and mutation rate at the locus of interest. We assigned each variable a high value and a low value based on current literature and ran a total of 16 simulations using a full factorial experimental design, altering only one variable at a time. This allowed us to study variables independently and assess how they affect the frequency of the migrant allele over time. We compare the impact of each variable by holding other variables constant and comparing the frequencies of the migrant allele.

We assigned high and low values for the four parameters based on actual events of historic admixture (Table 1). The high value for migrating population size was set at 1000, indicative of a large group like the Norse in the North of America[10]; the low value was set at 40, a generic number that could represent any small group of migrants, either in a boat or a migrating family. The high value for native population size was set at 40,000, the size of a large Mayan city in 1492; the low value was 1000, the size of a small city at the same time[35]. The high value for the number of generations since the migratory event was 174 generations ago, roughly the time of the ancient Lemba migration to Africa[36]; the low value of 44 generations represents the recent Norse migration[10]. Our simulations represent migration events that have occurred relatively recently (in the past 3,000 years), and the results should be interpreted accordingly. Although one may be able to extrapolate our results to more distant admixture events, additional simulations could better illustrate these scenarios.

**Table 1:** Simulation variables

Variable	Low Value	High Value	Source
Migrant Population Size	40	1,000	[10]

Native Population Size	1,000	40,000	[35]

Generations Ago	44	174	[10,36]

Mutation Rate	0.0043	1.3 × 10^-8^	see Table 2

The high and low values for mutation rate were chosen based on the mutation rates of the regions of the genome that are used in current genetic research. Determining which regions are preferred in genetic studies is a difficult question, as there are many possibilities; the literature involving just the human migration to America contains (but is not limited to) studies performed using autosomal genes[37], autosomal microsatellites[38], Y chromosome[26], mtDNA[39,40], and SNPs[38]. To determine the mutation rates used in our simulations, we chose a high and low value among these genomic regions (Table 2). In our simulations, we used a high mutation rate of 0.0043 mutations/locus/generation and a low rate of 1.3 × 10^-8 ^which represent mtDNA and autosomal loci, respectively.

**Table 2:** Mutation rates

Genome Region	Mutation Rate	Source
Autosomal	2.5 × 10^-8^	[48]

Y Chromosome	3 × 10^-3 ^to 1 × 10^-8^	[49,50]

X Chromosome	1 × 10^-8^	[51]

Microsatellites	4.5 × 10^-4^	[52-55]

mtDNA control region	4.3 × 10^-3^	[56,57]

Common regions of the human genome used in genetic research and their mutation rates.

In the first simulations, we modeled only one locus per individual and assumed no recombination; one locus is adequate to assess the role of these parameters on allele frequencies. We also initialized the migrant population with the migrant allele fixed (all migrant individuals possessed the migrant allele). This is unrealistic, but provides a best-case scenario for detecting the migrant allele. We replicated each simulation 250 times.

The second simulation category was a single simulation designed to mimic the genetic landscape of a true admixed population. We assigned mid-range values for migrant population size (200), native population size (5,000), and generations (100). In order to more realistically model a current study, we followed 1,000 loci on 20 different chromosomes on each individual. This represents a sample much larger than the recommended number needed in order to detect large human admixture[41]. A standard recombination rate of 1.26 cM/Mb was used[42], though the human recombination rate has been shown to be negligible over 100 generations[42]. At the beginning of the simulation, an initial migrant allele frequency and a mutation rate were randomly generated for each of the 1,000 loci on each individual, in order to model the DNA seen in actual human genetics research. The methods of random generation are outlined below.

**Figure 1:**
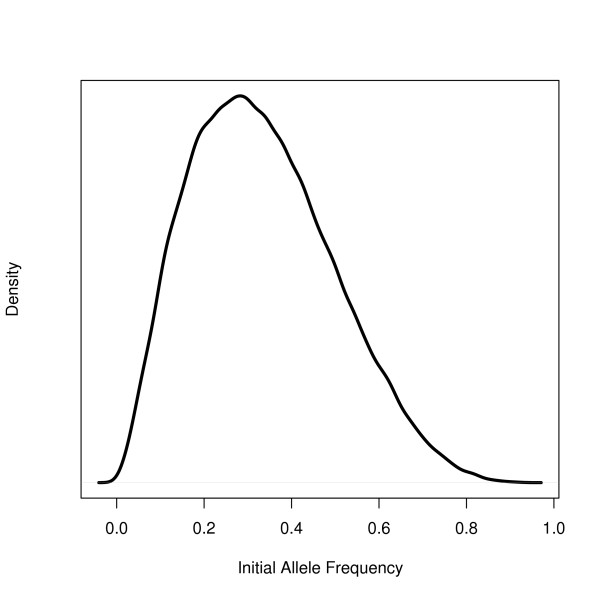
**Initial allele frequencies**. Density of a Beta distribution with a mean of 0.3 and a standard deviation of 0.17. Initial allele frequencies for all alleles were randomly generated from this density.

Initial allele frequency is difficult to assign because of the variability of allele frequencies in the human genome. Alleles with frequencies less than 5% are considered rare but are the most common categorization of SNPs and some alleles demonstrate frequencies greater than 90% (though these common alleles are rarely used in genetic studies)[43]. While the majority of SNPs are found in the 5% range, we built a simulation that will provide the best-case scenario for finding migrant alleles. Accordingly, we chose a much larger level for the average of initial allele frequencies, 30%. To generate frequencies in this range, we used a Beta distribution with a mean of 0.30 (Figure 1) and assigned a random frequency to each migrant locus. We also assumed that the migrant alleles were all absent in the native populations.

**Figure 2:**
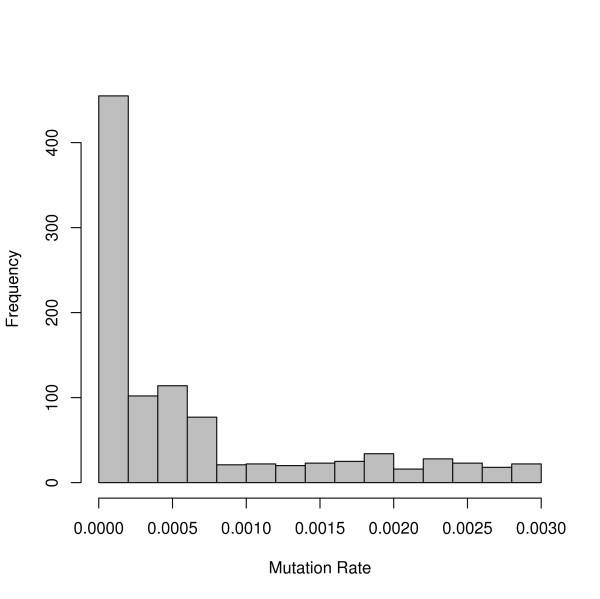
**Mutation rates**. Histogram demonstrating the distribution of mutation rates randomly assigned to the 1,000 simulated loci.

Mutation rates depend on the region of the genome used in a study. Differing mutation rates in the literature were presented earlier (Table 2). There is no estimate for which region of the genome is used most often in genetic studies; we, therefore, drew random values that capture the entire distribution of mutation rates seen in today's literature. For this simulation, we drew mutation rates equally from three different uniform distributions: one representing autosomal DNA with a low mutation rate (1 × 10^-9^, 1 × 10^-6 ^mutations/locus/generation), one representing microsatellites and some sex chromosomes (1 × 10^-6^, 7 × 10^-4^), and one representing mtDNA (1 × 10^-5^, 3 × 10^-3^) (Figure 2). We followed the migrant allele frequency at each locus through 100 generations. Final analyses and graphs were completed using the R software.

### Sample size study

In order to understand what must be done to successfully study data from historic admixture, we constructed a sample size study using the data from simulation 2. Small human genetics studies test approximately 50 loci when studying populations[44]. Given the calculated frequency of migrant alleles in our simulated population, we calculated the number of migrant alleles that would be seen, on average, in each human subject of a genetic study. This is accomplished using the cumulative density function (CDF) of a binomially distributed random variable where the size parameter is 50 and the probability parameter is the expected migrant allele frequency. In comparison, one of the larger human genetic studies to date sequenced 993 loci in each human subject[45]. Accordingly, we followed the same protocol to investigate a study of this magnitude, using the binomial CDF with a size parameter of 993 and the same probability parameter.

The most recent studies have again raised the bar as far as loci per subject, sampling 650,000 loci in each individual[25]. Although sampling more loci will find a larger number of migrant alleles, the proportion of such markers in the population does not change when more samples are taken. The study conducted by Li et al. (2008) samples about 20 individuals per population group, a number similar to previous studies. Accordingly, we investigated the sample size necessary to find at least one migrant allele at each of the loci sequenced in a large genetic study.

## Results

### Simulation study 1

**Figure 3:**
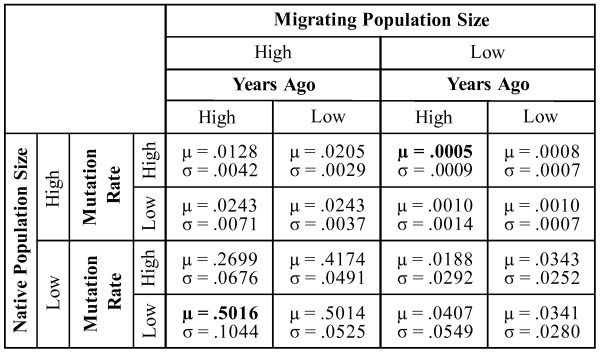
**Simulation results**. The probability of detecting historic, migrant alleles under all combinations of 4 essential genetic parameters. The average final migrant allele frequency of 250 replications of each parameter set is reported as the mean (μ) frequency of migrant alleles. The standard deviation (σ) of the 250 replications is reported for each parameter set below the corresponding mean. The two parameter sets with the highest and lowest mean allele frequencies are in bold.

**Figure 4:**
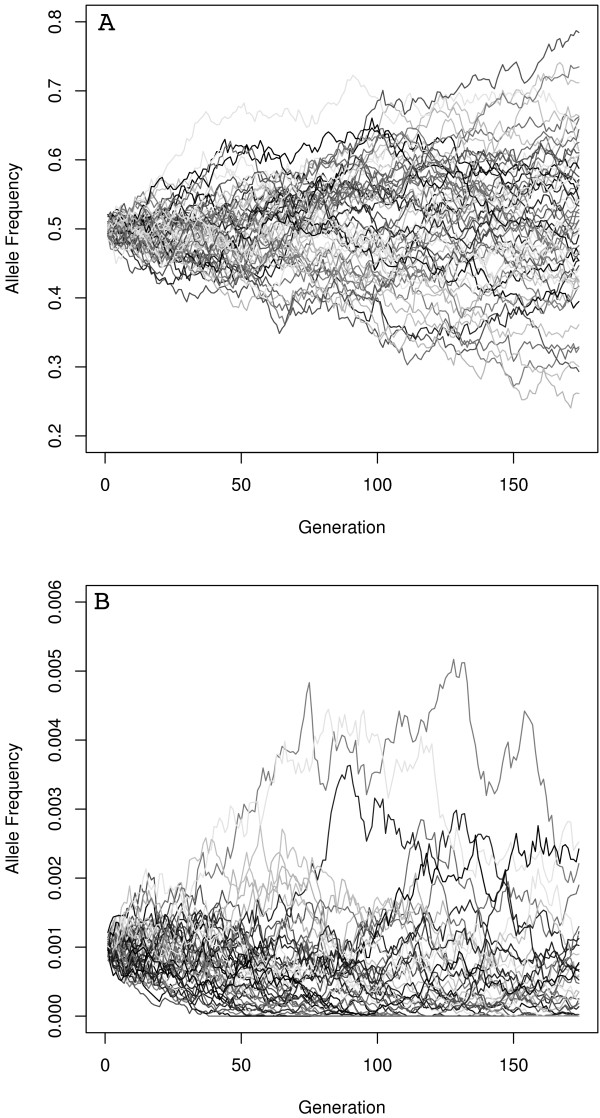
**Genetic drift**. Individual replications of the parameter sets highlighted in Figure 3. The migrant allele frequency of each replication at each generation are reported and plotted in line format, each replication is described by a single line. Only a sample of 50 replications was used, as 250 lines would be difficult to distinguish. The first parameter set is characterized by an initial allele frequency of 0.5 while the second parameter set has an initial migrant allele frequency of less than 0.001. Genetic drift and mutation cause the allele frequencies to change over time, resulting in some allele extinction and an overall distribution of frequencies at the end of the simulation.

**Figure 5:**
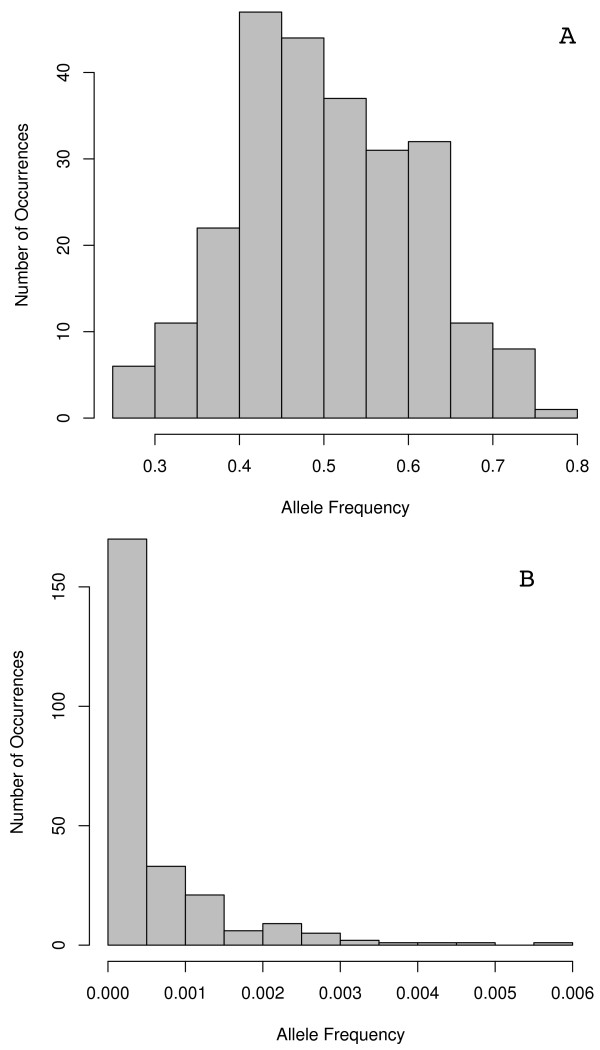
**Distribution of final allele frequencies**. Histogram of the final allele frequencies recorded over 250 replicates in the parameter sets highlighted in Table 2. These histograms are a representation of the last recorded allele frequencies from Figure 4. They demonstrate the distribution of the migrant allele frequency expected to be found in today's population, given the assumed genetic parameters (A: large migrant population, small native population, low mutation rate, and more distant admixture advent. B: small migrant population, large native population, high mutation rate, and a more distant admixture advent)

We calculated the frequency of the migrant allele at the final generation in each of the 16 simulations. The mean and standard deviation of this frequency, among the 250 replicates, are reported for each of the 16 simulations (Figure 3). We found that the parameter set that led to the highest mean allele frequency value included a low native population size, high migrant population size, and low mutation rate and was unchanged by the time since the migration event. The parameter sets that led to the lowest mean allele frequency value were: high native population size, low migrant population size, high mutation rate, and high number of generations (highlighted in Figure 3). For these two parameter sets, we randomly selected a fifth of the 250 replicates to illustrate the stochasticity of genetic drift (Figures 4 and 5). For the parameter set with the highest final mean allele frequency, we found replicates with final allele frequencies as low as 25.5% or as high as 78.5%. This parameter set also had the highest standard deviation (.1044), indicative of the wide range of final values in the different replicates. For the parameter set with the lowest final mean allele frequency, many of the replicates drifted to extinction (45.6%), while the highest allele frequency was 0.006%.

### Simulation study 2

**Figure 6:**
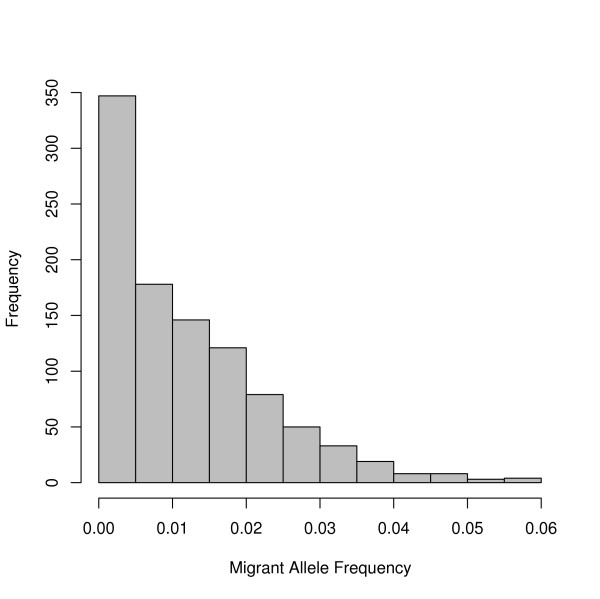
**Distribution of final allele frequencies**. A histogram showing the migrant allele frequencies found at 1,000 loci in a generic simulated population. This histogram illustrates the allele frequencies one would expect to find if 1,000 informative alleles were sampled from a current population that experienced admixture circa 2,000 years ago, given that the population had the specified genetic parameters.

For the second simulation study, we followed 1,000 loci through a simulation that could represent a human population (of 5,000 individuals) that experienced admixture (of 200 individuals) circa 2,000 years ago. Out of the 1,000 simulated loci, 140 (14%) drifted to extinction within 100 generations (Figure 6). These extinct alleles, combined with the effects of mutation, decreased the expected allele frequency of the final generation to 1.017%, a 16% decrease from the original value.

### Sample size study

**Figure 7:**
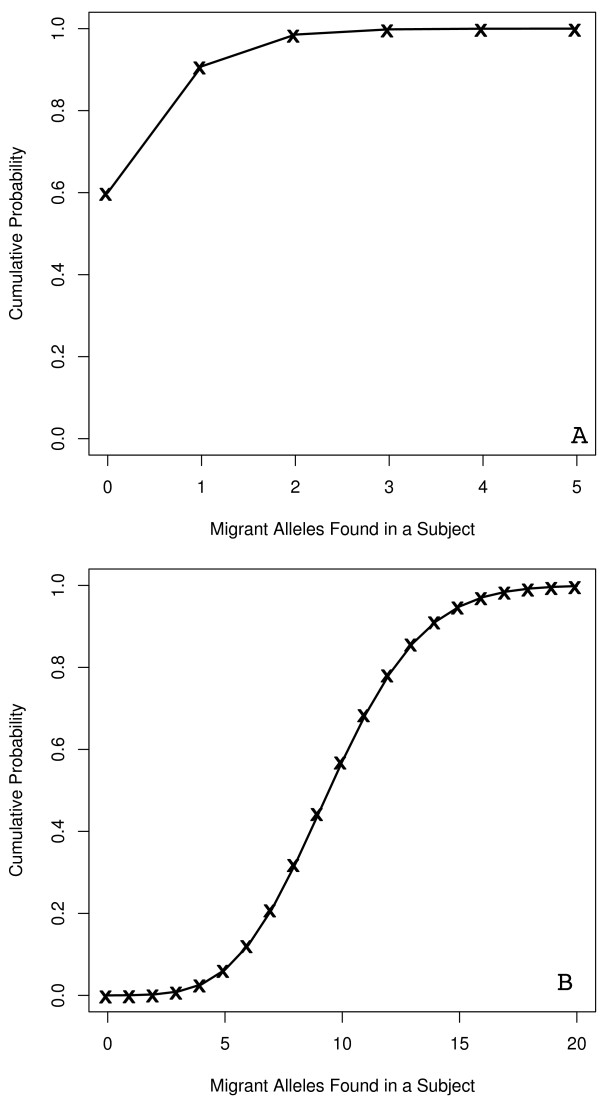
**Migrant allele expectation CDFs**. The CDF functions for the number of migrant alleles expected to be found in an admixed population for a study sampling 50 loci from each subject (A) and a study sampling 993 loci from each subject (B) (with an expected migrant allele frequency of 1.017%).

The average final allele frequency of the migrant allele in our population from the second simulation was 1.017%. We calculated the cumulative density function (CDF) for a genetic study that samples 50 loci for each individual and where the probability of detecting the migrant allele is equal to the probability found in our simulations. The CDF demonstrates that in 60% of individuals sequenced for 50 loci, we would not expect to find a single migrant allele (Figure 7a). Furthermore, we will only find more than one migrant allele in 9% of the subjects examined.

In the case of a large study with as many as 933 loci, based upon the expected migrant allele frequency of 1.017%, almost every subject would demonstrate at least one migrant allele (Figure 7b). In fact, most subjects would demonstrate more than 9 migrant alleles. However, while large studies would expect to succeed in finding more migrant alleles in today's population, this alone cannot link the admixed population to the migrant population. The migrant alleles will still only represent, on average, 1% of every allele sequenced in the entire study. Therefore, although 9 migrant alleles may, on average, be found in each subject, it is hard to know if the migrant alleles will be redundant among loci and subjects or spread evenly throughout all the loci in the study. Additionally, these numbers could be considerably lower depending on the allele frequency in the migrating population.

## Discussion

Our results provide some important insights in detecting historic admixture. The simulations we present illustrate the effect that initial parameters have on the outcome of human admixture. Simple adjustments in the parameters in our simulation series changed the expected allele frequency outcome from as low as 0.0005 to over 0.50, an increase of three orders of magnitude. The results of any admixture study using genetic data, then, are highly dependent on the variables presented in these simulations (e.g., mutation rate, population sizes, and time since admixture (number of generations)).

High mutation rates can decrease the expected migrant allele frequency and the variability by more than 50 percent, especially in populations that experienced earlier migrations. For example, an increased mutation rate can change the mean final allele frequency from .0243 to .0128, or from .5016 to .2699 (depending on other variables, as reported in Figure 3). Researchers should keep this in mind when selecting loci for analysis. Because some DNA mutation rates are highly variable, choice of locus can have a profound impact on the number of migrant alleles detected years later. Many studies advocate the use of mtDNA due to data collecting feasibility and other factors. However, because the mutation rate is generally higher in mtDNA, it could corrupt signal in studies addressing historic admixture, even when the time frame is relatively recent.

The sizes of the migrant and native populations are fundamental for an understanding of expected allele frequency. With time since admixture as low as those we consider in our simulations, the most important factors are the sizes of the migrating and native populations. In our simulations, if the native population is large, changing the migrating population size results in a change of mean final allele frequency from .0243 to .0010. If the native population is small, those numbers change to .5016 and .0407. These are the most significant differences illustrated by our simulations and they attest to the important role of population sizes. Researchers should not expect to find many alleles from a small migratory group of 50 individuals in a large population today, even if sampling methods are exhaustive.

Additionally, we see that time plays an important role. The standard deviations presented in Table 1 demonstrate that allelic frequencies vary widely, particularly as the number of generations increases. High mutation rates combined with large time spans can reduce migrant allele frequencies significantly. When the mutation rate is low, however, the time since admixture does not affect the final mean allele frequency much (or at all), but it still has a profound impact on the standard deviation. For example, a change in time since admixture in one parameter set almost doubles the standard deviation from .0525 to .1044. As time increases, genetic drift causes the spread of final allele frequencies to increase, particularly when the population sizes are small. Thus, as the time since the admixture event increases, sample size for both loci and subjects becomes increasingly important.

In our second simulation, most of the migrant alleles are present in less than 2% of the population. In a study of a population where few subjects from many human populations are studied, alleles from a small-scale admixture will usually not be recovered at all. And these rare alleles could easily be ignored in favor of haplotypes that better categorize the population into clusters.

Our results demonstrate a profound and general fact: the values of these genetic parameters can drastically alter the expected frequency of migrant alleles in today's populations. Even in our simulations, where steps have been taken to ensure a best-case scenario for the migrant allele, there is often a large spread of possible outcomes. DNA data have been touted as a panacea for recovering information about the past, but their use depends so extensively on factors that are beyond our control that their application is not always appropriate. It is imperative, therefore, that researchers understand the implications of the variables we have presented and not rely solely on DNA sequence data when researching small, recent human migrations. We can only hope to understand basic details of population history when quantifying genetic data and even valid results derived from genetic data may still be misleading if viewed unilaterally, as demonstrated by Harpending et al [46,47].

Our results, however, are not completely ominous. Carefully designed studies should be able to draw specific and valid conclusions from genetic data. One area for major improvement is the number of individuals and loci sampled. Our results indicate that a large sample size and large number of loci are needed to obtain robust results. Studies that are unable to sample sufficiently do not have the power to draw appropriate conclusions and should be interpreted with caution. Our results give guidelines for a variety of conditions and allow researchers to analyze the benefits of increasing sample sizes given their populations of interest. Because of the real possibility that a certain allele will have drifted to extinction, even sampling 100% of a population at a single locus may not reveal a single migrant allele, even if it was fixed in the migrant population. If one is faced with the challenge of researching small-scale admixture, it is necessary to identify migrant alleles even if they show up in a very small proportion of loci and subjects. Consequently, phylogenetic methods must be created that can pinpoint very small similarities between populations. Table 3 summarizes the genetic and experimental factors that we believe will increase the chance of detecting admixture in today's populations. One complication that arises in such situations, however, is that very recent migration and admixture will further complicate the results. Identifying migrant alleles that are rare will be very difficult, not only because of the increased sampling necessary to detect them, but because of the noise that is likely to be introduced in the time since the event under examination.

**Table 3:** Improving probability of detecting historic admixture

**Genetic Parameters**	**Experimental Design**
• Large Migrant Population	• Identify informative migrant alleles
• Small Native Population	• Test large number of loci
• Low mutation rate at loci of interest	• Large sample size for each population
• Fewer generations since admixture event	• Establish methods for detecting rare alleles
	• Collaborative approach (Archeology, Anthropology, Linguistics)

Perhaps most importantly, it must be remembered that drift is stochastic and that historic genetic parameters are, for the most part, unknown. Thus, the absence of specific genetic data is not conclusive evidence against historic admixture. Our results illustrate several parameter sets that would cause admixture to be either completely or practically undetectable today. To address the inconsistent results found in DNA all but the largest genetic studies need to continue to consider anthropologic, archeological, and linguistic data in order to formulate conclusions. Finally, our study demonstrates the utility of simulation studies to put bounds on parameter values and sample sizes for studies of human migration events.

## Conclusion

The ability to detect historic admixture and make correct inferences based on genetic data depends on the interplay between population sizes, mutation rates, time, and other parameters. We explore the parameter space of historic alleles in current populations and demonstrate the broad implications of each of these genetic parameters on modern allele frequencies. Our results provide guidelines with respect to the population genetic parameters and their values needed to detect migrant alleles in an admixed population. While studies that focus on large admixture events should be able to draw specific and valid conclusions, we suggest that genetic data be used with caution when studying small admixture events. The random nature of admixed genetic data seen in these simulations demonstrates that the utility of genetic data is dependent on the context of each individual study. Increasing the number of loci and the number of individuals sampled will increase the probability of detecting small traces of signal, but other sources of evidence should always be considered where possible.

## Authors' contributions

AS and NCS designed the simulations. NCS wrote and ran the simulations using simuPOP. AS analyzed and formatted the resulting data in R. AS and NCS wrote the manuscript. KAC conceived the study and provided expertise and advice throughout the process, including critical comments on simulation design and on the manuscript.
